# Molecular co-localization of multiple drugs in a nanoscopic delivery vehicle for potential synergistic remediation of multi-drug resistant bacteria

**DOI:** 10.1038/s41598-022-22759-z

**Published:** 2022-11-07

**Authors:** Amrita Banerjee, Dipanjan Mukherjee, Arpan Bera, Ria Ghosh, Susmita Mondal, Subhadipta Mukhopadhyay, Ranjan Das, Hatem M. Altass, Sameer. S. A. Natto, Ziad Moussa, Saleh A. Ahmed, Arpita Chattopadhyay, Samir Kumar Pal

**Affiliations:** 1 Department of Physics, Jadavpur University, 188, Raja S.C. Mallick Rd, Kolkata, 700032 India; 2 Technical Research Centre, S. N. Bose National Centre for Basic Sciences, Block JD, Sector III, Salt Lake, Kolkata, 700106 West Bengal India; 3 Department of Chemical and Biological Sciences, S. N. Bose National Centre for Basic Sciences, Block JD, Sector 3, Salt Lake, Kolkata, 700106 India; 4 Department of Chemistry, West Bengal State University, Kolkata, 700106 India; 5 Chemistry Department, Faculty of Applied Science, Umm Al-Qura University, Mecca, 21955 Saudi Arabia; 6 Physcis Department, Faculty of Applied Science, Umm Al-Qura University, Mecca, 21955 Saudi Arabia; 7 Department of Chemistry, College of Science, United Arab Emirates University, Al Ain, P.O. Box 15551, Abu Dhabi, United Arab Emirates; 8 Department of Basic Science and Humanities, Techno International New Town Block, DG 1/1, Action Area 1 New Town, Rajarhat, Kolkata, 700156 India; 9 Department of Physics, Sister Nivedita University, DG 1/2 New Town, Action Area 1, Kolkata, 700156 India

**Keywords:** Biochemistry, Chemical biology, Microbiology, Chemistry, Materials science, Nanoscience and technology

## Abstract

Anti-microbial resistant infection is predicted to be alarming in upcoming years. In the present study, we proposed co-localization of two model drugs viz., rifampicin and benzothiazole used in anti-tuberculosis and anti-fungal agents respectively in a nanoscopic cationic micelle (cetyl triethyl ammonium bromide) with hydrodynamic diameter of 2.69 nm. Sterilization effect of the co-localized micellar formulation against a model multi-drug resistant bacterial strain viz., Methicillin resistant *Staphylococcus aureus* was also investigated. 99.88% decrease of bacterial growth in terms of colony forming unit was observed using the developed formulation. While Dynamic Light Scattering and Forsters Resonance Energy Transfer between benzothiazole and rifampicin show co-localization of the drugs in the nanoscopic micellar environment, analysis of time-resolved fluorescence decays by Infelta-Tachiya model and the probability distribution of the donor–acceptor distance fluctuations for 5 μM,10 μM and 15 μM acceptor concentrations confirm efficacy of the co-localization. Energy transfer efficiency and the donor acceptor distance are found to be 46% and 20.9 Å respectively. We have also used a detailed computational biology framework to rationalize the sterilization effect of our indigenous formulation. It has to be noted that the drugs used in our studies are not being used for their conventional indication. Rather the co-localization of the drugs in the micellar environment shows a completely different indication of their use in the remediation of multi-drug resistant bacteria revealing the re-purposing of the drugs for potential use in hospital-born multi-drug resistant bacterial infection.

## Introduction

World Health Organization (WHO) has declared antimicrobial-resistant (AMR) infections as one of the top 10 global public health threats^1^. It was reported that in 2019 alone, the AMR organisms were the primary cause of 1.27 million deaths worldwide^2^. If corrective mechanisms are not put into place, it is likely to skyrocket. In an alarming report, commissioned by the UK government, it was predicted that antimicrobial resistance may cause up to 10 million deaths annually by 2050^3^. Amongst antimicrobial-resistant bacteria, the emergence of multidrug-resistant (MDR) bacteria species is of great concern, primarily because of their ability to mutate genes and to reduce drug action. Poor drug binding capacity and low cell penetration^4^ make multidrug-resistant bacteria responsible for almost 65% infections which are linked with HealthCare-Associated disease^5^. The inappropriate use of antibiotics is responsible for rise in bacterial resistance, which makes the clinical management of infections hard to manage with conventional antibiotics^6^. Numerous bacteria, especially MDR, can grow and survive on the contaminated damp surfaces^7^, for a longer time, mainly in healthcare facility. The dirt and organic element from human or animal origin can work as substrates for the growth of the bacteria and facilitates the spread of micro-organism induced infection from individual to the community. Hence, the rapid and effective disinfection of surfaces and environmental cleanliness is one of the primary measures to control the spread of MDR bacteria. Amongst all multidrug-resistant bacteria, Methicillin-resistant *Staphylococcus aureus* (MRSA) has been categorized as a high priority multidrug-resistant pathogen by WHO^8^. It can cause a mild skin infections to fatal infections^6^. MRSA is resistant to penicillin, methicillin, oxacillin and amoxicillin^9^. MRSA, being one of the most critical human organism and colonizing bacteria, is responsible for one of the leading causes of infections worldwide^10^. Infections induced by MRSA carry significant clinical load for the patient and medical practitioner^11^. Prolonged hospital stay, increased hospital cost as well as higher hospital mortality^12^ also impart a huge economic burden to the patient-family, healthcare systems and to the society^13,14^.

Emerging MDR strains of MRSA are clinically notorious because of their lack of response to conventional antimicrobial therapy. It is known that biofilm formation is one of the main causes behind antibiotic resistance. Biofilm, an organized colony of microorganisms, forms an extracellular polymeric matrix of substance and shows adhesion with the neighbouring living and non-living surfaces^15^. It facilitates microorganism interaction and protect tolerant cells from the change in living environment for a long period of time^16^. The biofilm matrix strengthen the tolerance to disinfectants by wrapping the underlying cells^17,18^ and by restricting diffusion of disinfectants into the biofilm matrix^19^. Hence, regulating biofilm formation has become an alternative approach to limit persistent infections^20^. Regular use of disinfectants on potential-contaminated surfaces have become an important routine practice for sterilization^21,22^ as it reduces biofilm formation and reduces transmission of infectious pathogens, in turn. Currently, bleach (sodium hypochlorite)—derivative solutions are used in healthcare facilities^23^ as disinfectant spray. Although, it has significant adverse effect on bacterial contaminants, due to its oxidizing capacity and toxicity, the bleach-derivative products are not suitable for long-term use^24^. Despite the presence of significant environmental threat, chlorine (Cl_2_) and its derivative sodium hypochlorite (NaOCl) are still the most widely used disinfectants. Organic chloride compounds released by these chloride-based sterilising agents in the wastewater are toxic for aquatic organisms and possess a significant threat as environmental contaminants^25^. Hence, new avenues are required to be examined to control the huge adverse impact of the MRSA infections^26^.

Previously it was pointed out that the combination of two different drugs linked through a selectively chosen drug carrier, improves the efficacy of the system against AMR activities^27,28^. The present study explores the co-localization of an antituberculosis drug rifampicin (RF) with another drug benzothiazole (BT) in an organized assembly of Cetyl Triethyl Ammonium Bromide (CTAB) micelles, and their prospect as an antibacterial spray against MRSA. RF is a popular drug with a proven safety profile for treatment of tuberculosis for its sterilizing activity and ability to shorten treatment^29,30^. BT is an important class of medicine possessing antibacterial^31^, antiviral^32^, anticancer^33^, antifungal^34^ and antitumor^35^ properties. On the other hand, CTAB solution which works as a nanoscopic vehicle by bringing BT and RF together, too is safe and familiar for their antimicrobial properties^36^. In this context it is worth mentioning that some studies already investigated the development and design of novel drug delivery systems using density functional theory calculations and molecular dynamics simulations^37,38^.

For our study, to obtain precise location of the drugs RF and BT in the organized assembly of CTAB, FRET between BT (energy donor) and RF (energy acceptor) was employed because the fluorescence spectrum of BT overlaps reasonably well with the absorption spectrum of RF. Time-resolved studies on FRET^39–41^ were employed to obtain precise information on localization of the drugs (RF and BT) in CTAB micelles. In addition, dynamic light scattering (DLS) technique was used to investigate the integrity of the nanoscopic organized assembly of CTAB micelles. Furthermore, we have applied a kinetic model developed by Tachiya to analyze the fluorescence decay of BT in presence of RF in CTAB micelles^42,43^ and compared them with the experimental data. These findings are finally extended to MRSA bacteria and the promising potential of the RF-BT encapsulated in CTAB micelles as a strong antibacterial spraying agent has been established. It was observed that separately these compounds, i.e., RF, BT and CTAB show low to moderate level effectivity against the MRSA colonies. Whereas their combined effect has shown impressive results by successfully eliminating almost all the colonies of MRSA. Singular and doublet combinations of these chemicals were also studied to comprehensively explore sterilising possibilities. Computational biology study on the formulation shows that the drugs rifampicin and benzothiazole, although conventionally used as tuberculosis and antifungal agents, can be repurposed for remediation of the MDR micro-bacteria including MRSA.

## Materials and methods

### Chemicals

2-(2-Hydroxyphenyl) benzothiazole (BT), rifampicin (RF), Cetyltrimethylammonium bromide (CTAB), Ethanol, acetonitrile (ACN), Dimethyl sulfoxide (DMSO) were purchased from Sigma Aldrich, California. All the chemicals were used as purchased without any further purification. Luria broth (LB) and LB top agar medium for bacterial studies were bought from HIMEDIA.

### Sample preparation

50 mM, 3 mM and 4 mM stock solutions of CTAB in DI water (Milli-Q), RF in ethanol and BT in acetonitrile were prepared. The solutions were diluted according to the experimental study.

### Photophysical studies

Dynamic Light Scattering (DLS) measurements were performed in a Nano S Malvern (Zeta-seizer) instrument employing a He–Ne laser source (wavelength = 632.8 nm) of 4 mW power and equipped with a thermostatic sample chamber. All the scattered photons were collected at 173**°** scattering angle. The scattering intensity data were processed using the embedded instrumental software to obtain the hydrodynamic diameter (d_H_) and particle size distribution of the scatterer in each sample. The instrument measures the time-dependent fluctuation in the intensity of light scattered from the particles in solution at a fixed scattering angle. The intensity autocorrelation function of the time-dependent fluctuation in scattered intensity gives estimation of the hydrodynamic diameter (d_H_) of the clusters. Hydrodynamic diameter d_H_ is defined by

1 $${d}_{H}=\frac{{k}_{b}T}{3\pi \eta D}$$

where, k_b_ is the Boltzmann constant, η is the viscosity of solvent (here, 50 mM CTAB), *T* is the absolute temperature, and *D* is the translational diffusion coefficient. The resolution of the measuring instrument in this study is 0.65 nm.

### Steady state and time-resolved fluorescence spectroscopy

Steady state absorption and emission spectra were measured with Shimadzu UV-2600 spectrophotometer and Jobin Yvon Fluorolog fluorimeter, respectively. All the picosecond resolved fluorescence transients were measured by using commercially available time-correlated single-photon counting (TCSPC) setup with MCP-PMT from Edinburgh instrument, UK (instrument response function (IRF) of ~ 75 ps) using a 375 nm excitation laser source. All the fluorescence transients have been measured in magic angle. In our study, benzothiazole and rifampicin act as the donor and acceptor respectively. The non-emissive behaviour of the RF eliminates the possibility of the interference of RF in BT fluorescence transients in CTAB micelle. Details of the time resolved fluorescence setup have been discussed in our previous reports^44,45^.

### Fitting of picosecond resolved fluorescence transients

The observed fluorescence transients were fitted by using a nonlinear least-squares fitting procedure to a function

2 $$(X\left(t\right)=\underset{0}{\overset{t}{\int }}E(t{^{\prime}})R(t-{t}^{^{\prime}})dt{^{\prime}})$$

(*E*(t)) with a sum of exponentials (2) with pre-exponential factors (*B*_i_),

3 $$(R\left(t\right)=A+\sum_{i=1}^{N}{B}_{i}{e}^{-t/{\tau }_{i}}$$

With characteristic lifetimes (*τ*_i_) and a background (*A*). Relative concentration in a multiexponential decay is expressed as.

4 $${c}_{n}=\frac{{B}_{n}}{\sum_{i=1}^{N}{B}_{n}}\times 100$$

The amplitude-weighted average lifetime of a multiexponential decay is expressed as,

5 $${\tau }_{av}=\sum_{i=1}^{N}{c}_{i}{\tau }_{i}$$

6 $$\sum_{i=1}^{N}{c}_{i}=1$$

The quality of the transients fitting has been justified by observing reduced chi-square which is none other than the ratio of residual and weighted residual. In the transients fitting, the value of χ^2^ lies in between 1.0 and 1.2.

### FRET studies

In order to estimate the Forster resonance energy transfer efficiency from the donor (BT) to the acceptor (RF) and to determine the donor–acceptor pairs we have followed the methodology described in Lakowicz^46^. The critical donor–acceptor distance (R_0_) where the energy transfer efficiency is 50% was calculated using the formula below:

7 $${R}_{0}=0.211\times {[{\kappa }^{2}{n}^{-4}{Q}_{D}J\left(\lambda \right)]}^\frac{1}{6}$$

The refractive index (n) is considered to be 1.43. *k*^2^ is the orientational factor describing the relative orientation of the transition dipoles of donor and acceptor respectively in space. The orientational factor, *k*^2^ is mathematically related to the cosine of the orientational angle as follows:

$${\kappa }^{2}=(cos{\theta }_{T}-3cos{\theta }_{D}-cos{\theta }_{A})$$

where ${\theta }_{T}$ is the angle in between the transition dipole of the donor and acceptor respectively; ${\theta }_{D}$ and ${\theta }_{A}$ are the angle in between donor and acceptor dipole and the vector that joins the donor and acceptor dipole respectively. The donor (BT) and the acceptor (RF) are assumed to adopt all possible orientation during the energy transfer process for which the value of ${\kappa }^{2}$ is taken to be 0.667. Because, sixth root of the orientational factor is considered the maximum error introduced in determining the donor acceptor distance is not more than 30%. *Q*_D_ is the quantum yield of the donor in the absence of acceptor is measured to be 0.01 by considering quinine sulphate as a reference of Quantum Yield (QY) determination.

*J*(λ) is the overlap integral which signifies the degree of spectral overlap between the donor emission and the acceptor absorption and is expressed as:

8 $$J\left(\lambda \right)= \frac{\underset{0}{\overset{\infty }{\int }}{F}_{D}\left(\lambda \right){\varepsilon }_{A}(\lambda ){\lambda }^{4}d\lambda }{\underset{0}{\overset{\infty }{\int }}{F}_{D}(\lambda )d\lambda }$$

The D–A pair distance (${r}_{DA}$) can be calculated after getting the value of R_0_ from the following equation.

9 $${{r}_{DA}^{6}r}^{6}=\frac{[{R}_{0 }^{6}\left(1-E\right)]}{E}$$

*E* is the energy transfer efficiency calculated from the lifetimes of the donor in absence and in presence of the acceptor (τ_D_ and τ_DA_).

10 $$E=1-\frac{{\tau }_{DA}}{{\tau }_{D}}$$

${\tau }_{D}$ is the average lifetime of the donor and ${\tau }_{DA}$ is the average lifetime of the donor in presence of acceptor, obtained from the fitted parameters of the fluorescence transients.

The probability distribution, p(r), of the donor acceptor distances is calculated by nonlinear least square fitting procedure by using the following equation

11 $$p\left(r\right)=\{1/[\sigma -{\left(2\pi \right)}^\frac{1}{2}]\}\times \mathrm{exp}{\{-\frac{1}{2}\times [\left({r}_{1}-r\right)/\sigma]}^{2}\}$$

where r_1_ is the mean of the gaussian distribution function with a standard deviation σ, and r is the donor/acceptor distance obtained from FRET efficiency calculated by Eq. 10. Details of the calculation of the p(r) is described elsewhere^47^.

### Bacterial strain and culture conditions

The antibacterial action of the samples has been studied against a strain of MRSA bacteria. Methicillin-resistant *Staphylococcus aureus* (MRSA) strain (ATCC 25923) was procured from ATCC. For antibacterial assay, fresh MRSA bacteria have been cultured using Luria–Bertani (LB) medium in a shaker incubator at a temperature of 37 °C for 24 h. The freshly grown MRSA culture was further diluted 10^6^ times and samples were added. The treatment of bacteria was performed on LB agar plates by the colony-forming unit (CFU) assays method under dark condition. The cells were incubated with 1 mM of CTAB, RF (80 µM), BT (80 µM), RF-BT in 1 mM CTAB (The concentration of BT and RF is 80 µM), RF (80 µM) in 1 mM of CTAB and BT (80 µM) in 1 mM of CTAB separately for 3 h without any photo-activation. Then the cultures were uniformly spread on LB agar plates and the plates were incubated at 37 °C for 24 h to get the CFUs. To quantify the antibacterial activity, the CFU numbers were manually counted and presented as a bar diagram.

### Statistics

Data are represented as the mean ± standard deviation unless otherwise stated. Unpaired 2-tailed T-Test was used to calculate differences between groups. P < 0.05 was considered significant. For all statistical tests, GraphPad Prism (v8.0) software was used.

### Computational tools used to study compound-protein interaction

To predict Chemical-Protein (CP) Interaction Networks of the drugs on MRSA, the web-resource STITCH (version 5.0) provided by STITCH Consortium2016 (http://stitch.embl.de/) was used. STITCH database can predict about 430,000 chemicals and 960,0000 proteins curated from 2031 eukaryotic and prokaryotic genome^48,49^. The association for a chemical-protein interaction can be predicted by the confidence score, where higher score corresponds to a stronger interaction. Medium confidence score (0.4) was used for the present study. Eight different sources, i.e. experiments, text mining, neighborhood, gene fusion, databases, co-expression, co-occurrence and predictions are used to populate the active interactions.

## Results and discussions

**Figure 1:**
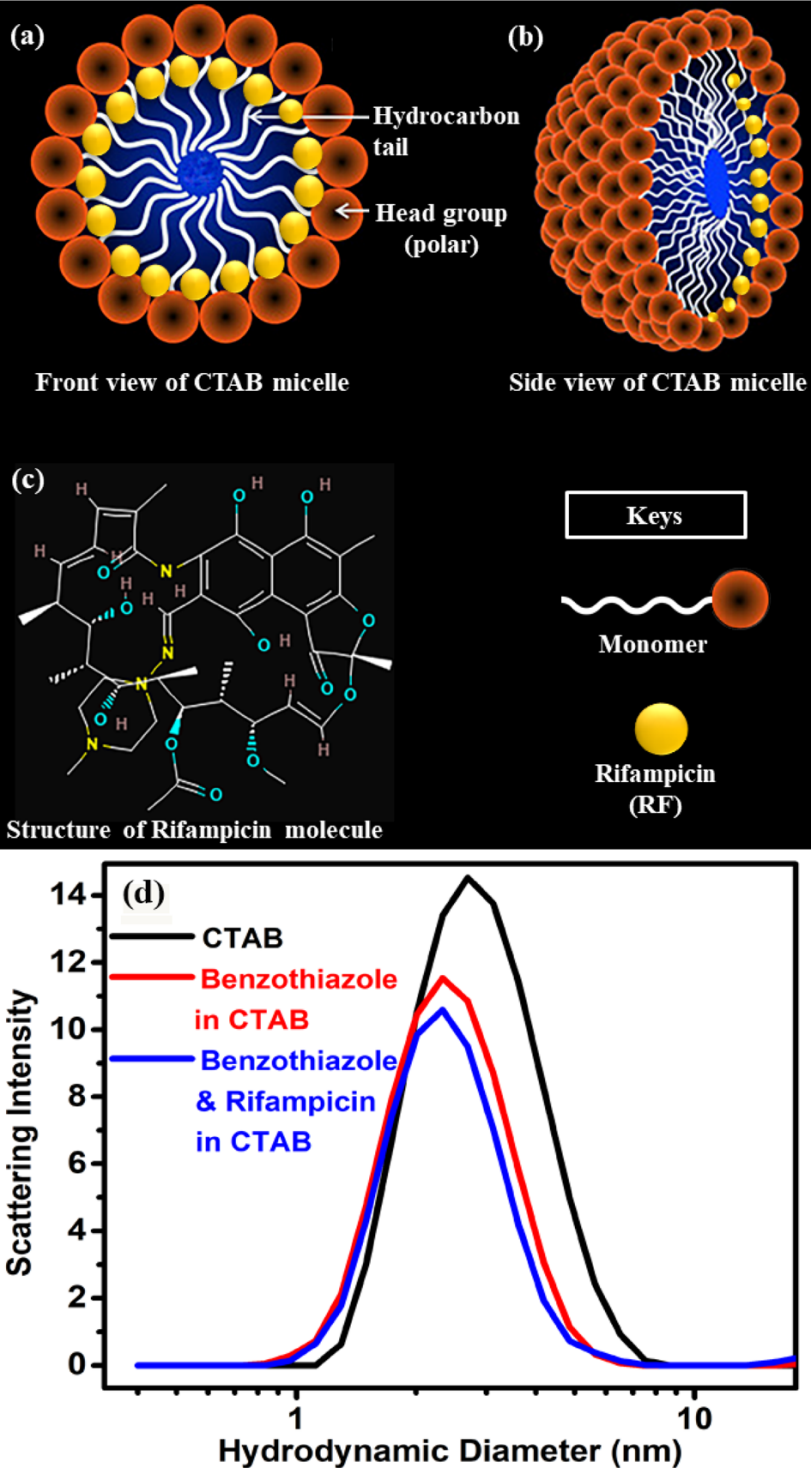
(**a**) Front view of CTAB micelle. (**b**) Side view of CTAB micelle. (**c**) Structure of rifampicin molecule. (**d**) Scattering Intensity of 50 mM CTAB micelle, BT added to CTAB and BT and RF added together to CTAB as obtained from the dynamic light scattering study.

The drug rifampicin is reported to be hydrophobic^50–52^, as it is less soluble in aqueous solution. However, in aqueous medium (pH = 7) RF can be amphiphilic or amphoteric^53,54^ considering its charge distribution. Thus. RF is expected to interact superficially with the cationic CTAB micelle or bind to the outer surface of CTAB micelle which is also amphiphilic^55^. Considering that RF at pH 7 remains in the Zwitterionic form^53^, it can interact electrostatically with the head group of the CTAB micelle and is therefore most likely to stay in the micelle–water interface^50^ as portrayed in Fig. 1a and b. Dynamic light scattering (DLS) studies (Fig. 1d) indicate that the CTAB micelles form mono-dispersed spheres, in aqueous buffer, of hydrodynamic diameter (d_H_) of 2.69 nm. After addition of the energy donor (BT) and the energy acceptor (RF) in 50 mM micellar solution, the hydrodynamic diameter peaks were observed to remain almost in the same position confirming our conjecture of binding of RF (Fig. 1c) inside the periphery of the cationic micelle. Except Dynamic Light Scattering (DLS), another technique is the HRTEM (High-Resolution Transmission Electron Microscopy) by which one can characterize the monodispersity of the micellar solution. Bahadur et. al. has already performed cryo-TEM at ~ 150 mM CTAB micellar solution and found it to be completely monodispersed which further discard the possibility of micellar aggregation in our study^56,57^. Another proof of the monodispersity is the UV–visible absorption of the drug loaded micelle where the existence of the aggregation can be ruled out by the higher absorption value of the drug at the absorption tail. Furthermore, our prepared micellar solution is completely transparent which further supports the monodispersity of the micellar solution at the particular concentration used in our study. The angstrom (Å) sized drugs should not bring any effect on the micellar size after encapsulation. The hydrodynamic diameter is calculated automatically by the instrument from the fitting parameter of the autocorrelation function. It is an average phenomenon and accuracy depends on the number of runs performed by an experimentalist during the experiment. Standard deviation of the Malvern DLS instrument in terms of hydrodynamic diameter lies within the range of ± 0.7 nm. In our study, the size reduction obtained as ~  ± 0.4 nm as shown in Fig. 1d, and to the best of our understanding it is inconclusive as it lies within fluctuation range of the measurement.

### Forster resonance energy transfer (FRET)

**Figure 2:**
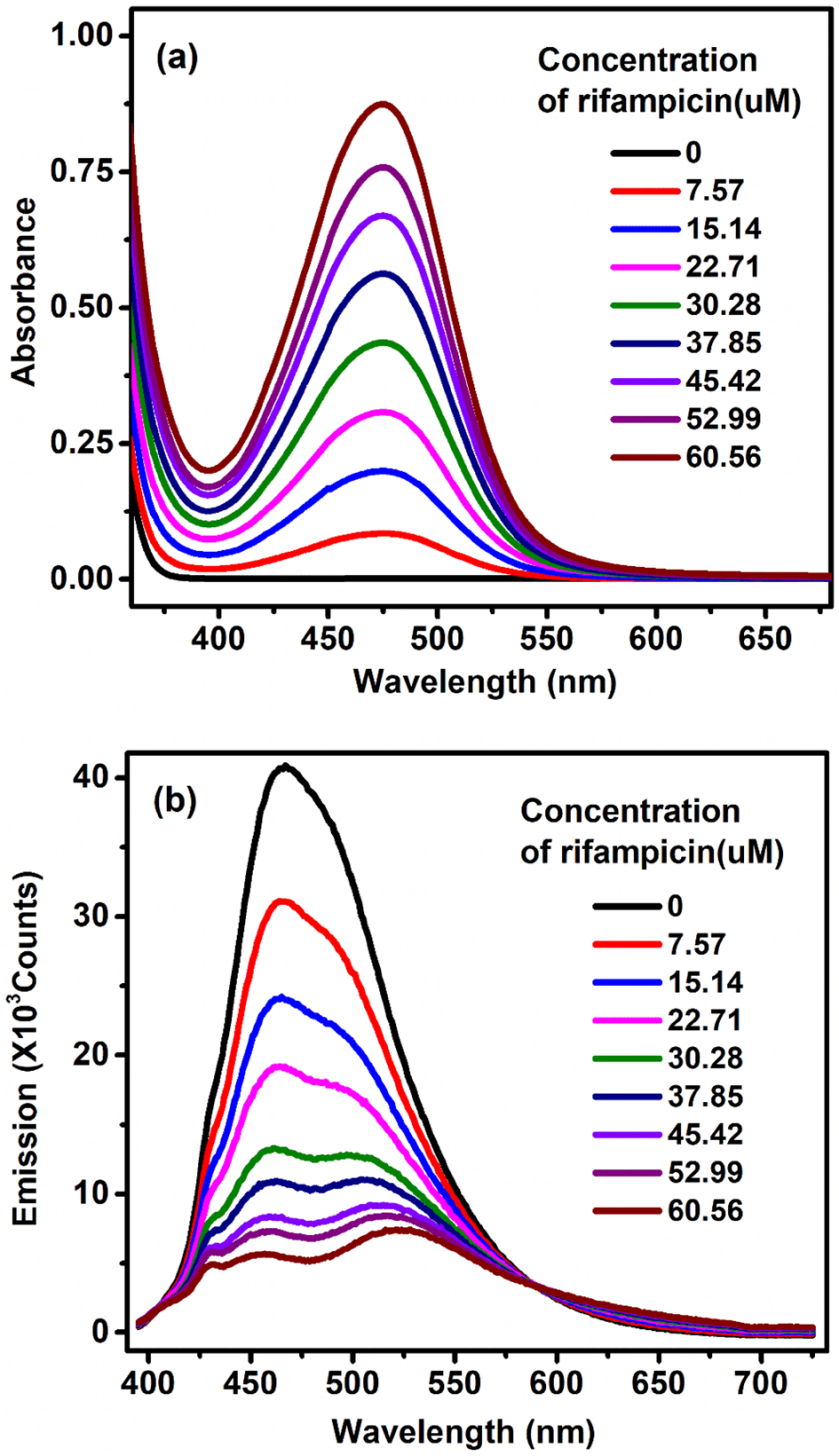
(**a**) Absorbance spectra of Rifampicin (RF) in 50 mM CTAB micelles for concentrations 0–60.56 µM. (**b**) Emission spectra of benzothiazole in 50 mM CTAB without and with varying concentrations of RF from 0 to 60.56 µM.

**Figure 3:**
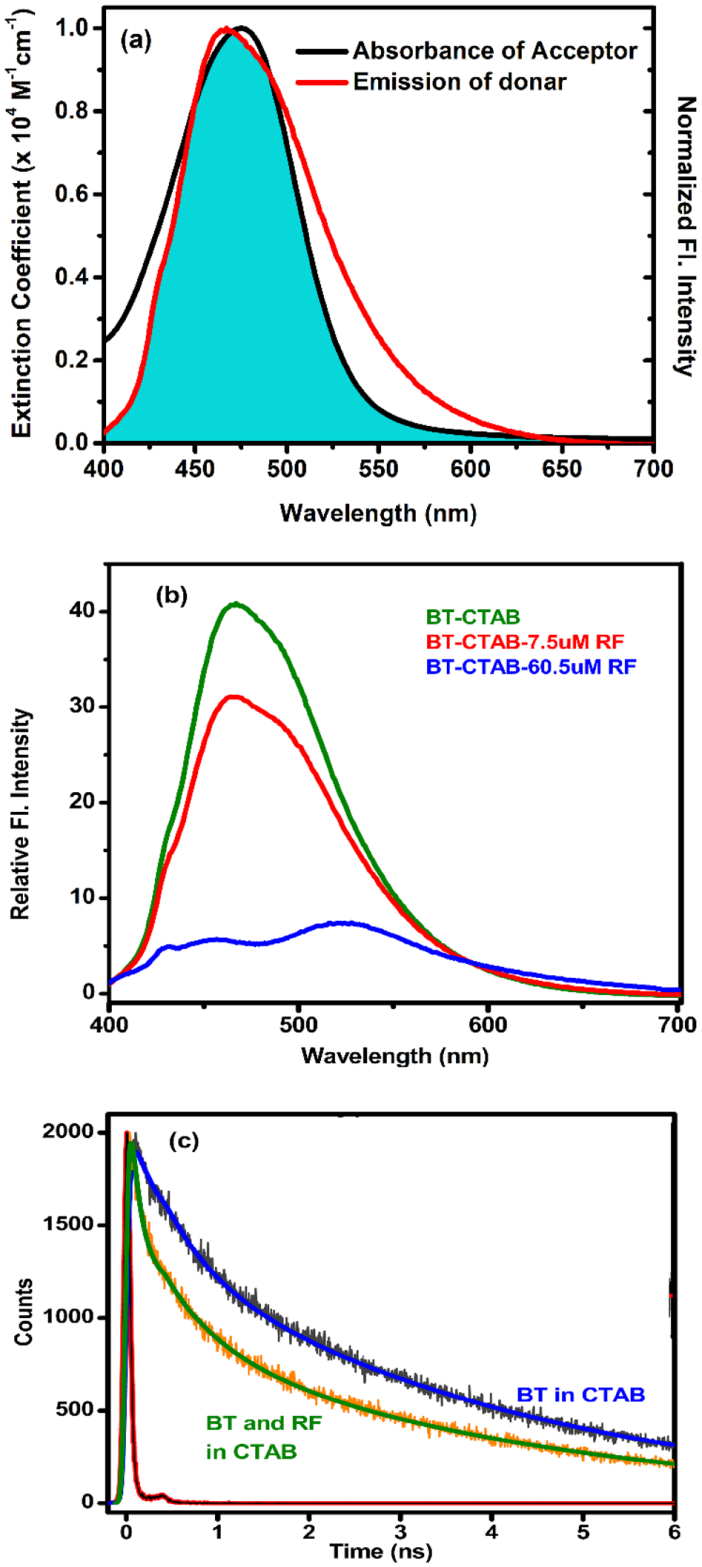
(**a**) Spectral overlap of BT fluorescence (Red) and RF absorbance (black) when both are incorporated in CTAB micelle. (**b**) Forster Resonance Energy Transfer (FRET) process is of BT ligand to RF within the CTAB micelle is evident from the quenching of the steady-state fluorescence intensity. (**c**) Picosecond-resolved fluorescence transients of the BT ligand as donor in CTAB micelle in absence and in presence of RF as an acceptor.

FRET is an energy transfer process between a pair of fluorophores, where the donor fluorophore, initially in its electronic excited state, transfers energy to an acceptor fluorophore^40,58^. In our study, RF, a well-known anti tuberculosis drug, is chosen as an acceptor for studying FRET in CTAB because of two reasons. Firstly, RF, being a neutral dye, localizes in the interfacial region of the micellar headgroup and hydrophobic hydrocarbon chains^59^. Secondly, the fluorescence spectra of BT as donor overlaps excellently (Fig. 3a) with the absorption spectra of RF (Fig. 2a). The drugs RF and BT were thus chosen due to their efficacy of forming FRET-pair. In order to study the co-localization of the two model drugs, FRET is found to be very efficient tool as indicated in our study. Figures 2b and 3b show significant decrease in fluorescence intensity of BT in 50 mM CTAB solution upon addition of increasing amount of RF indicating to an efficient energy transfer from BT to RF. Time-resolved fluorescence decay of BT becomes distinctly faster (Fig. 3c) in presence of RF consistent with significant quenching of the fluorescence transients of BT in CTAB micelle. The average fluorescence lifetime of BT decreases remarkably (Table 1) from 2.56 to 1.37 ns in presence of 10 μM of RF in 50 mM CTAB micelle confirming an efficient energy transfer from BT to RF which is consistent with a donor acceptor distance of 20.9 Å (Table 2). This highly efficient (E = 46%, Table 2) energy transfer from BT to RF is consistent with co-localization of both the drugs within the CTAB micelle.

**Table 1:** Time-resolved decay parameters of BT in CTAB in absence and in presence of RF at two different concentrations.

System	τ1/ns (%)	τ2/ns (%)	τ3/ns (%)	τavg/ns (%)
BT in CTAB	0.302 (30%)	1.263 (14%)	4.11 (56%)	2.56
BT in CTAB + 5 µM RF	0.209 (36%)	0.973 (18%)	4.07 (46%)	2.12
BT in CTAB + 10 μM RF	0.042 (54%)	0.550 (21%)	4.00 (25%)	1.37

**Table 2:** FRET parameters of BT (donor) and RF (acceptor) in CTAB micelle.

System	τD/ns	τDA/ns	E(%)	J (λ)/M(−1) cm(−1) nm(4)	rDA in Å	hw in Å
BT in CTAB + 5 µM RF	2.56	2.12	17	5.16 × 10^14^	26.6	2.23
BT in CTAB + 10 μM RF	2.56	1.37	46	5.16 × 10^14^	20.9	3.29

**Figure 4:**
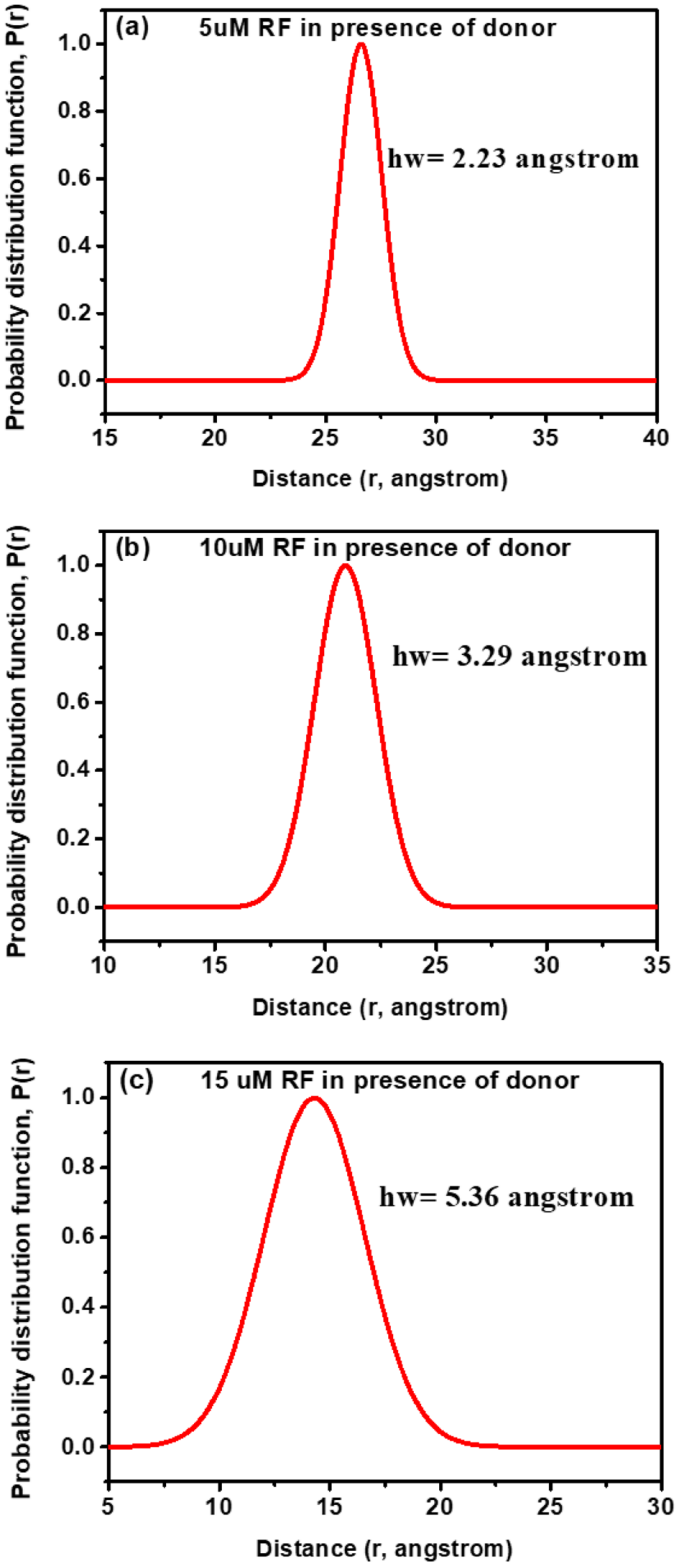
Probability distribution of donor − acceptor distances between Rifampicin and 2-(2-hydroxyphenyl)-benzothiazole in CTAB micelles for different concentrations of acceptor (**a**) 5 μM, (**b**) 10 μM, (**c**) 15 μM.

We have further employed the probability distribution (P(r)) of donor–acceptor distances to characterize the nature of the interaction of both the donor/acceptor at three different acceptor concentrations upon incorporation into the micellar cavity (Fig. 4). The distribution function becomes broader at higher acceptor concentration relative to that of the lower concentration, which corresponds to the higher average amplitude in fluctuation of the donor–acceptor distance during energy transfer process for the former than the later. This is further reflected in increasing value (Table 2 and Fig. 4) of the ‘hw’ parameter (full width at half maxima) of the P(r) upon increasing concentrations of the acceptor concentrations (RF). The increase in the amplitude of the donor/acceptor distance fluctuation is consistent with the progressive increase of the FRET efficiency and decrease in the donor/acceptor distance. Thus, the CTAB micelle turns out to be an efficient nanoscopic vehicle for co-encapsulation of both BT and RF, giving rise to a formulation with better antimicrobial activity than that based on only BT or RF.

### Infelta-Tachiya model for the quantitative estimation of the donor/acceptor distribution in the micellar cavity

For better assessment of the association between RF and BT in CTAB micelle, it is important to estimate the distribution of RF molecules, because, the energy transfer efficiency solely depends on the availability of the acceptor molecules in the vicinity of the donor molecules. Fluorescence transients of the fluorophores in CTAB micelle in the excited state can be considered by the Infelta − Tachiya kinetic model^42^.

12 $${P}_{n}^{*}\to {P}_{n}$$

13 $${P}_{n}^{*}\to {P}_{n}$$

Herein, *P*_n_^*^ signifies the CTAB micelle containing excited BT fluorophores, and n is number of the acceptor RF molecules. In contrast, P_n_ denotes the micelle which contains n number of quencher molecules in absence of any excited BT fluorophore. k_0_ is considered to be the total decay constant of the excited state donor fluorophore in absence of any acceptor molecules and k_q_ is the decay constant of the same fluorophore in CTAB micelle in presence of one quencher molecule. Subsequently the rate constant at which a donor molecule in micelle decays from an excited state in presence of ‘n’ number of quencher molecules can be expressed as k_0_ + nk_q_ and nk_q_ is the total energy transfer rate constant. In this particular kinetic model, it is postulated that the distribution of the quencher molecules around the vicinity of donor molecules follow the Poisson distribution^42^ which can be defined as

14 $$p\left(n\right)=\frac{{m}^{n}}{n!}\times exp(-m)$$

where m is the total average number of quencher molecules in micelles available to quench the BZ fluorescence. Mathematically m can be expressed as

15 $$m=\frac{{k}_{+}[A]}{{k}_{-}}$$

where, ${k}_{+}$ and ${k}_{-}$ are the associated rate constants by which an acceptor molecule enters the micellar cavity and exits from the cavity respectively. [A] is the concentration of the acceptor molecules in aqueous phase. By considering the above model, probability of finding the fluorophore molecules in its excited state at a particular time t can be expressed as

16 $${P}^{*}\left(t\right)={P}^{*}(0)\mathrm{exp}[-\left({k}_{0}+\frac{{k}_{0}{k}_{+}\left[A\right]}{{k}_{-}+{k}_{q}}\right)\times t+\frac{{k}_{q}^{2}{k}_{+}\left[A\right]}{{k}_{-}\times {\left({k}_{-}+{k}_{q}\right)}^{2}}\times \left\{1-exp\left[-{(k}_{-}+{k}_{q}\right)\times t\right]\}$$

For the case ${k}_{-}$<< ${k}_{q}$, the above equation reduces to,

17 $${P}^{*}\left(t\right)={P}^{*}(0)\mathrm{exp}\{-{k}_{0}t-m\times \left[1-exp \left(-{k}_{q}t\right)\right]\}$$

**Figure 5:**
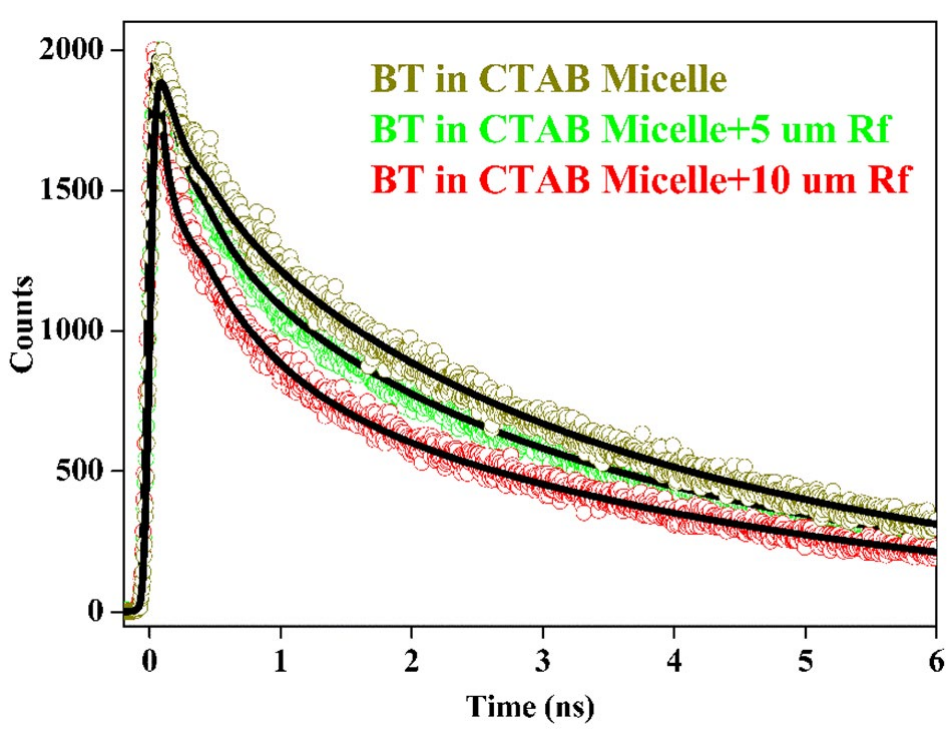
Time-resolved fluorescence transients of BT in CTAB in absence and in presence of two different concentration of RF. The bold line represents the fitting line of the curves by considering simplified infelta tachiya kinetic model of donor acceptor association in CTAB micelle.

Figure 5 displays the fluorescence transients of BT in CTAB micelle in absence and in presence of various concentrations of RF (acceptor). The transients are fitted by using Eq. (17) based on the Infelta-Tachiya model of the excited state decay of the fluorophore. Those transients are well fitted to an exponential function ($X\left(t\right)={\int }_{0}^{t}E\left({t}^{^{\prime}}\right)\times P\left(t-{t}^{^{\prime}}\right){dt}^{^{\prime}})$ where it is deconvoluted during the fitting procedure with respect to instrument response function (IRF) (by using Micromath Scientist). It is evident that the transients are fitted reasonably well (Fig. 5) by the Infelta-Tachiya model with reduced χ^2^ value between 1.0 and 1.2. From the fitting parameters (Table 3) it becomes evident that availability (m) of the RF molecules in the vicinity of the energy donor drug BT increases in CTAB micelle at increased concentration of RF. The energy transfer rate constant (k_q_) of BT in CTAB micelle also increases with increasing concentration of acceptor (RF) indicating to closer association between BT and RF, which is consistent with the results obtained from FRET. However, conventional FRET analysis fails to provide overview of the distribution of the acceptor molecules in the donor vicinity in a nanoscopic micellar system quantitatively. The average number of acceptor (RF) molecules to quench the fluorescence of a single BT molecule is found to be < 1 (m = 0.3, Table 3) even when excess concentration of RF is used, which discards the possibility of quenching of the donor (BT) fluorescence thorough the association of multiple acceptor molecules in the micellar cavity. Our observation is quite similar to that of^60^ where the fluorescence of CDS QDs has been quenched by Ox170. The average number of attracted acceptor towards the trap state of QDs is more or less 1, which indicates that the energy transfer process in the particular event occurs at a single molecule level of both donors/acceptors. Banerjee et. al. has further studied acceptor association induced quenching of donor transients in nanoscopic micellar surface by a potential carcinogen benzo[a]pyrene (BP) as donor in presence of various acceptors^61^ which shows the available number of acceptor molecule in micellar surface to quench donor fluorescence is ~ 1 for a FRET efficiency of ~ 86%.

**Table 3:** Values of quenching parameters obtained from simplified infelta tachiya model.

System	${k}_{0}$/(ns)	$m$	${k}_{q}$/(ns)
BT in CTAB + 0 μM RF	0.17		
BT in CTAB + 5 μM RF	0.17	0.21	0.74
BT in CTAB + 10 μM RF	0.17	0.37	1.03

### Antibacterial studies

**Figure 6:**
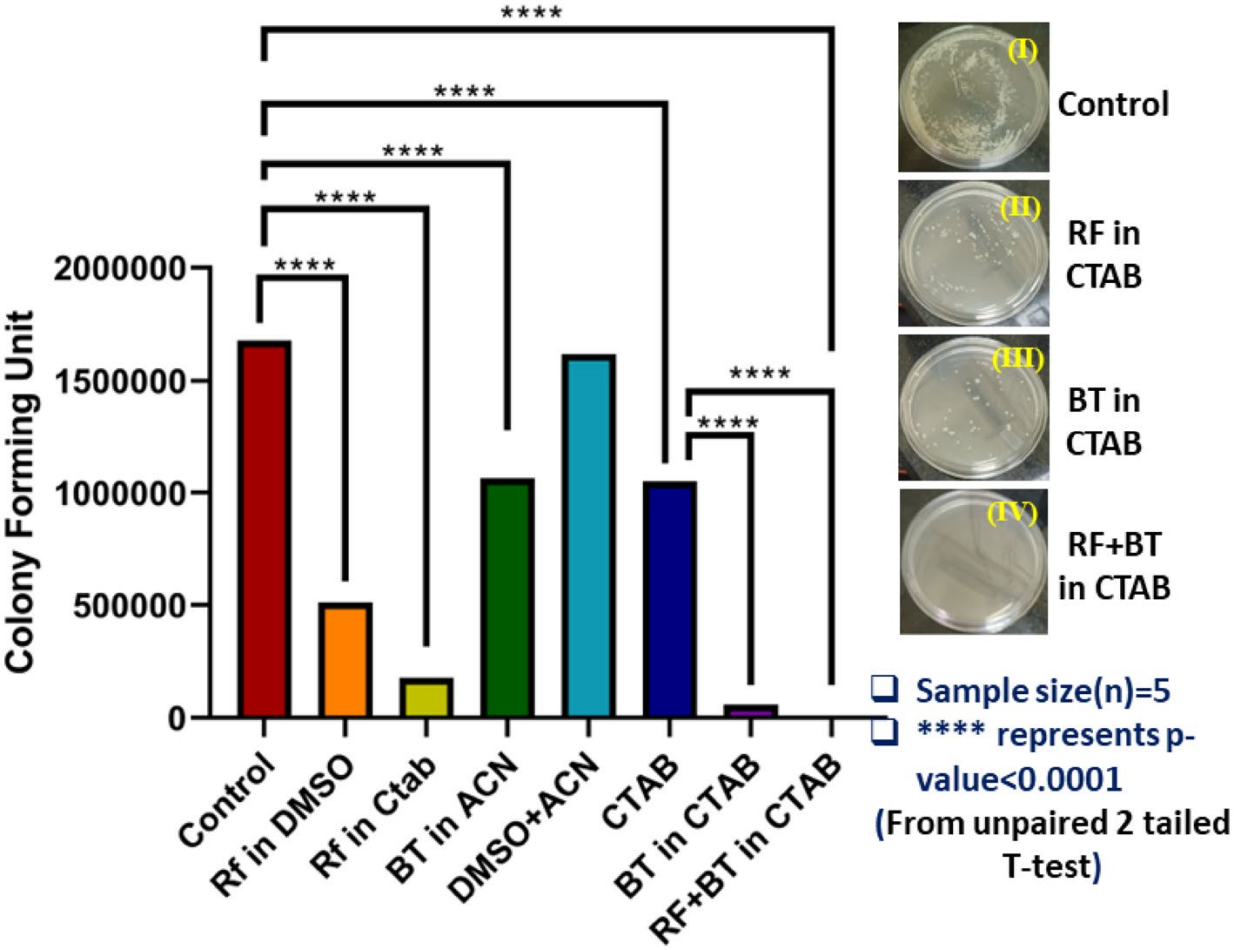
Bacterial viability studies after treatment with RF-BT complex in CTAB (2 units), RF in CTAB (178 units), BT in CTAB (59 units), BT in ACN (1064 units) and RF in DMSO (512 units) where the control is 1677 units. The inset shows images of MRSA culture plates for (I) Control, (II) Treated with RF in CTAB, (III) Treated with BT in CTAB and (IV) Treated with RF-BT in CTAB. The sample size is 5(n) for each group. p-value for each group is < 0.0001 as indicated by ‘****’ obtained from unpaired two-tailed T-test (see text); p < 0.05 was considered to be significant.

The antimicrobial activity of co-localized rifampicin-benzothiazole (RF-BT) in the nanoscopic system of a CTAB micelle was investigated against the MRSA growth to explore the sanitization potential against bacterial infections. To probe the antibacterial action of RF-BT in CTAB, RF in CTAB, BT in CTAB and CTAB only were used for incubating the culture for 3 h. As shown in Fig. 6, almost no colonies were observed (the bacterial growth is found to have decreased by 99.88% in CFU) for the RF-BT complex in CTAB. The bacterial growth is found to be decreased by 37.33% in CFU for CTAB only. In case of RF and BT, the bacterial growth is found to be decreased by 69.46% and 36.55% in CFU respectively. On the other hand, a huge decrement of the bacterial growth is observed for RF in CTAB (decreased by 89.38% in CFU) and BT in CTAB (decreased by 96.46% in CFU) separately. The anti-bacterial study of RF-BT in aqueous medium has not been performed as both the drugs show instability in aqueous solution and result in precipitation. The individual effects of RF in DMSO and BT in acetonitrile are explored along with the combined effect of RF and BT in CTAB.From these results it is evident that CTAB itself is an antibacterial agent and is efficient as a drug delivery vehicle for BT and RF which triggers an overall huge antibacterial effect.

For studying the drugs loading level, the drug solution (RF and BT) was prepared in PBS buffer, separately. Then the solution was dropwise added to the CTAB solution and incubated at room temperature followed by vigorous stirring for ~ 6 h. Free drug solution was removed by centrifuging the CTAB micelle encapsulated drug at 14,000 rpm for ~ 50 min. The absorbance of encapsulated drug (A_encapsulated_) was obtained by using UV–visible spectrophotometer after dissolving the pellets obtained after centrifugation in PBS buffer at respective absorption maxima of the drugs. The encapsulation efficiency (EE) was found to be 70% calculated by following equation^62^:

18 $$EE \left(\%\right)=\frac{{A}_{encapsulated}}{{A}_{total}}\times 100$$

where A_total_ was the absorbance of drug added to the solution before centrifugation.

However, in the present work, our intension is to establish the synergistic effect of two co-localized drugs in a nanoscopic delivery vehicle for potential remediation of a multi-drug resistant bacteria. The details of the ratiometry of the drugs on the anti-bacterial effect including MED (Minimum Effective Dose) will be the subject of our future publication.

There are other methods to test bacterial viability like fluorescence microscopy-based assay by PI (Propidium Iodide) staining^63^ or FACS (fluorescence-activated single cell sorting, a flow cytometry technique)^64^. However, in the present work, our aim is to establish the synergistic effect of two co-localized drugs in a nanoscopic delivery vehicle for potential remediation of a multi-drug resistant bacteria. Thus, we did not focus on multiple methods and followed a single standard method which is well reported in the literature to study the viability of bacteria^65,66^.

To study the effect of the drugs rifampicin and benzothiazole in CTAB micelle, bacterial cultures were performed 5 times for each group (control, RF in DMSO, RF in CTAB, BT in ACN, CTAB, BT in CTAB, RF + BT in CTAB etc.) and their difference was calculated to identify their significance level. The sample size for each of the groups were 5. The p-value was calculated using unpaired 2-tailed T-Test and p < 0.05 was considered to be significant. The ‘*’ represent p-value < 0.1 and ‘****’ represent p-value < 0.0001^67^.

### Computational rationalization of anti-microbial effect

#### Synergism amongst CTAB, RF and BZ

We have observed during the study that separately the compounds, RF, BT and CTAB show low to moderate level effectivity against the MRSA colonies whereas their combined effect has shown impressive results by successfully eliminating almost all (99.88%) the colonies of MRSA. The extraordinary effect of RF, BT and CTAB together may be hypothesized using predictive biological interactions.

**Figure 7:**
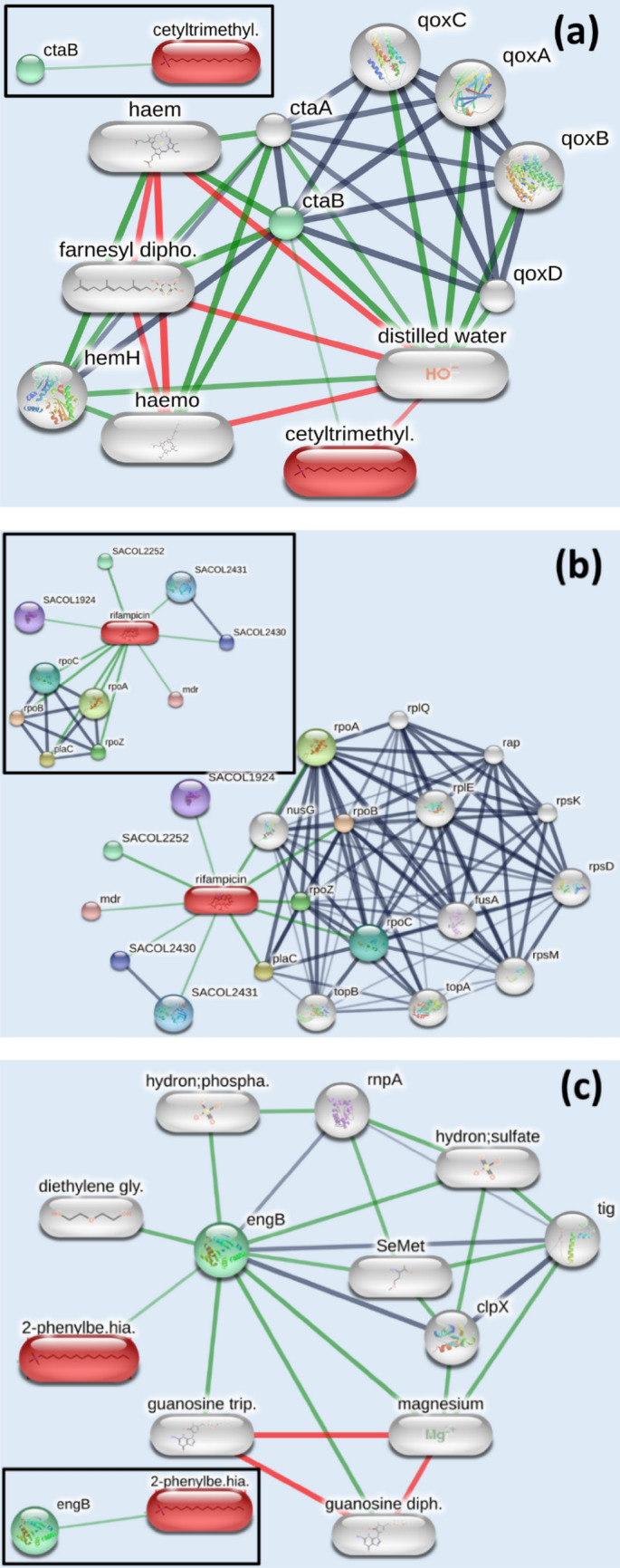
Direct and indirect compound protein interactions of (**a**) CTAB (**b**) Rifampicin (RF) and (**c**) Benzothiazole (BT) on MRSA. The inset for each shows the direct interactions only.

Figure 7a–c depict compound-protein (CP) interactions for all three compounds, viz, CTAB, RF and BT separately using STITCH 5.0. Primary as well as secondary interactions were considered for CP network analysis.

To grasp the mechanism of action of CTAB, rifampicin and benzothiazole on MRSA, three separate comprehensive tables (Tables 4, 5 and 6) of target proteins/compounds and their biological activities are listed below.

**Table 4:** Effect of cetyltrimethyl ammonium bromide (CTAB) on MRSA.

Name of protein/compound	Mode of action	Biological function
ctaB	Direct	Protoheme IX Farnesyltransferase (ctaB) controls the hemolytic activity in MRSA^68^ which in turn responsible for virulence of *S. aureus*^69^. CTAB decreases virulence activity, as well as enhanced pigment production and persister survival of MRSA^68^ which justifies the low effectiveness of CTAB against MRSA
ctaA	Indirect	CTAB alters the production of enzyme ctaB, which also affects the generation of cytochrome oxidase assembly protein ctaA. ctaA is a key enzyme which controls not only its starvation survival, but also, recovery, and Cytochrome Biosynthesis in *Staphylococcus aureus*^70^
qoxA, qoxB, qoxC, qoxD	Indirect	Quinol oxidase subunits, qoxA, qoxB, qoxC, qoxD genes^71^ play an important role in respiration and folding of membrane proteins^72^. Although presence of persister cells favored by the terminal oxidases may lead to chronic infection^73^
hemH	Indirect	Heme synthesis is directly related to iron metabolism. hemH gene catalyzes the heme biosynthesis, which influences the addition of ferrous iron (Fe2+) into protoporphyrin IX. Both porphyrins and iron facilitate the generation of highly reactive oxygen species, which is toxic to the cells and may lead to damage of most biomolecules^74^
farnesyl diphosphate	Indirect	High concentration of farnesyl diphosphate, slows down the production of the protein heptaprenyl diphosphate synthase, which is responsible for menaquinone formation, a key electron transporter in many bacteria^75^

**Table 5:** Effect of antituberculosis drug Rifampicin (RF) on MRSA.

Compound	Mode	Activity
SACOL1924	Direct	Potential multi-drug exporting ABC transporter permease/ATP-binding protein^76^
SACOL2252	Direct	AcrB/AcrD/AcrF family protein and part of multidrug resistance pumps^77^
Mdr- SACOL0700, SACOL2430, SACOL2431	Direct	ABC transporter ATP-binding protein/permease
plaC, SACOL1618	Direct	RNA polymerase sigma factor which facilitates the binding of RNA polymerase to specific initiation sites. It was observed that dispute in the plaC gene cause fatal effect on *S. aureus*^78^
rpoZ, SACOL1222	Direct	DNA-directed RNA polymerase subunit omega which facilitates RNA polymerase assembly. Mutation in rpoZ gene leads to stress resistance and to fully form a biofilm^79^.
rpoA, SACOL2213	Direct	The RNA polymerase (RNAP) core enzyme of *Staphylococcus aureus* comprises of four different subunits, which are encoded by the rpoA, rpoB, and rpoC genes, respectively. This DNA-dependent RNAP activates the transcription of DNA into RNA
rpoB, SACOL0588		
rpoC, SACOL0589		Rifampin prohibits the production of RNAP and attaches to the β-subunit of RNAP within the DNA/RNA channel and hampers the elongation of RNA^80^
rplQ, SACOL2212	Indirect	The rplQ gene encodes the ribosomal protein L17 of the 50S subunit^81^
nusG, SACOL0582	Indirect	nusG is a transcription regulation protein which controls transcription elongation, termination and antitermination^82^
topB, SACOL2243	Indirect	DNA topoisomerase III, encoded by the topB gene, releases the supercoiling and torsional tension of DNA. It may have a pivotal role in bacterial genome stabilization. Mutants with topB deletion have a higher rate of spontaneous deletions^83^
topA, SACOL1267	Indirect	DNA topoisomerase I, encoded by the topA gene, releases the supercoiling and torsional tension of DNA, which is important for DNA repair^84^
rpsM, SACOL2215	Indirect	*rpsM gene regulates* translation/ribosome structure. Its mutations may prompt the stress response and develop enhanced homogeneous resistance^85^
fusA, SACOL0593	Indirect	mutations in fusA leads to high proportion of *S. aureus* colonizing on skin and nares and are resistant to fusidic acid^86^
rpsD, SACOL1769	Indirect	30S ribosomal protein S4, one of the major rRNA binding proteins is also known as ribosomal protein small-subunit D (rpsD). It may be useful as a target^87^
rpsK, SACOL2214	Indirect	30S ribosomal protein S11, necessary for growth and reproduction^88^
rplE, SACOL2227	Indirect	50S ribosomal protein L5 which binds the 5S RNA into the large ribosomal subunit, and forms part of the central protuberance
rap, SACOL2236	Indirect	50S ribosomal protein L2 and one of the primary rRNA binding proteins. Clinical isolates of *S. aureus* are responsive to RAP (RNAIII-activating peptide), which facilitates biofilm formation capacity and virulence^89^

**Table 6:** Effect of benzothiazole (BT) on MRSA.

Compound	Mode	Activity
engB, SACOL1720	Direct	Ribosome biogenesis GTP-binding protein YsxC is necessary for regular cell division and for the maintenance of normal septation. It may also be involved in ribosome biogenesis^90^
rnpA, SACOL2739	Indirect	The protein component of ribonuclease P (RNase P), rnpA is responsible for mRNA decay and may also have a role in bulk mRNA turnover. This makes it a suitable target for antimicrobial drug discovery^91^
clpX, SACOL1721	Indirect	ATP-dependent protease ATP-binding subunit ClpX. In MRSA, clpX is an essential element which governs virulence^92^
tig, SACOL1722	Indirect	trigger factor involves in protein export in a mouse model of infection. tig gene mutation led to reduced biofilm development but no substantial decrease in virulence^93^
diethylene glycol	Indirect	Poly(ethylene glycol) coatings are familiar for reduction in microbial adhesion by numbers and binding strength^94^
guanosine diphosphate guanosine triphosphate	Indirect	They help bacteria to adjust according to different environmental stresses^95^
Magnesium	Indirect	Mg2+ metal ions rupture *S. aureus* membranes and kill stationary-phase *S. aureus* cells, which denotes membrane-activity^96^
SeMet	Indirect	SeMet may inhibit bacterial growth and is protective on macrophages^97^

From the above-mentioned protein descriptions, it is quite evident that the combined application of CTAB, RF and BT creates a synergistic effect, as the pathway of action of these three compounds are totally different. The results from antibacterial study are also consistent with computational observations.

## Conclusion

In this study, a highly potential antibacterial agent is developed by embedding a well-known antituberculosis drug rifampicin with a common antifungal drug benzothiazole inside a cationic CTAB micelle as delivery vehicle. Dynamic light scattering (DLS) studies have been utilized to characterize the integrity of a CTAB micelle and to confirm the encapsulation of RF and BT within the micelle. FRET measurements along with probability distribution of donor–acceptor distances were employed to locate the binding of the drug RF with respect to BT in the micellar surface. The distance between the donor (BT)–acceptor (RF) pair is found to be 20.9 Å in the micellar surface. Infelta-Tachiya model has been further employed to understand the kinetics of energy transfer from BT to RF molecules with increasing quencher (RF) concentration, The results obtained it are consistent with the FRET data. The credence of this antibacterial agent has been finally established by anti-bacterial studies on MRSA bacteria. Predictive biological models also support the MRSA eradication mechanism. These studies demonstrate the high potential of a new antibacterial sanitizing spray for use in healthcare facilities against multi drug resistant MRSA bacteria, responsible for numerous health hazards.

## Data Availability

The datasets used and/or analyzed during the current study are available from the corresponding author on reasonable request.
